# Revisiting the Nutritional Mode of *Floccularia luteovirens*: A Case for Facultative Saprobic Capacity

**DOI:** 10.3390/life16020287

**Published:** 2026-02-07

**Authors:** Siyuan Gou, Xu Zhao, Yanqing Ni, Tongjia Shi, Zhiqiang Zhao, Lihua Tang, Wensheng Li, Yan Wan

**Affiliations:** 1 College of Food and Biological Engineering, Chengdu University, Chengdu 610106, China; 2 Institute of Urban Agriculture, Chinese Academy of Agricultural Sciences, Chengdu 610299, China; 3 Chengdu National Agricultural Science and Technology Center, Chengdu 610299, China; 4 Zhuoni County Agricultural Technology Extension Station, Gannan 747600, China; 5 The Edible Fungi Research Institute of Shanghai Academy of Agricultural Sciences, Shanghai 201403, China; 6 Key Laboratory of Coarse Cereal Processing, Ministry of Agriculture and Rural Affairs, College of Food and Biological Engineering, Chengdu University, Chengdu 610106, China

**Keywords:** *Floccularia luteovirens*, mycorrhizal relationship controversy, facultative nutrition, carbohydrate-active enzymes (CAZymes), artificial domestication

## Abstract

*Floccularia luteovirens* is a rare and edible fungus endemic to the Qinghai–Tibet Plateau. Traditional viewpoints have inferred it to be a mycorrhizal fungus based on its spatial association with *Kobresia*, yet direct morphological evidence (e.g., Hartig net) and molecular evidence is lacking. Through a systematic review of the existing literature, this study found that all current evidence supporting a mycorrhizal relationship is merely indirect inference. In contrast, experiments conducted by our research team demonstrated that this fungus colonizes well on sawdust-based substrates, which is compatible with saprobic growth capacity and does not exclude the possibility of conditional mycorrhizal symbiosis in natural environments. Based on these findings, we propose that *F. luteovirens* may adopt a facultative nutritional mode to adapt to the alpine environment. Genomic analysis revealed that the CAZyme repertoire of *F. luteovirens* (including key enzyme families such as GH6, GH7, and AA1) shows high similarity to that of the saprobic fungus *Agaricus bisporus* and appears to be more comprehensive than that of the ectomycorrhizal fungus *Boletus edulis*, based on current annotation data. This pattern suggests its potential capacity for lignocellulose degradation. The successful cultivation of its closely related species *Lepista sordida* on various lignocellulosic substrates further supports this functional potential. This study proposes that *F. luteovirens* employs a ‘facultative nutrition’ strategy, which presents an alternative perspective to the traditional view of obligate dependence on mycorrhizal symbiosis. These findings contribute to our understanding of fungal adaptation in alpine environments and may inform strategies for artificial domestication of this valuable species.

## 1. Core Controversy and Proposal of the Hypothesis

*Floccularia luteovirens* is a superior edible and medicinal fungus nurtured under the unique geographical and climatic conditions of the Qinghai–Tibet Plateau. As an important biological resource of the plateau [1], it is recognized as one of the most valuable wild fungi for development and utilization.

*F*. *luteovirens* grow exclusively in herbaceous vegetation, mainly in alpine meadows, alpine steppes, and other vegetation types. Due to its formation of fairy rings in pastures and the discovery of hyphae attached to the roots of forage grasses, Zhou Qiming first proposed a symbiotic relationship between *F. luteovirens* and *Kobresia* based on these phenomena in 1984 [2]. However, with subsequent citations and descriptions by researchers, reports have directly stated that “this fungus is a mycorrhizal fungus [3].” This assertion, however, lacks conclusive direct evidence. Notably, current scholars generally agree that whether this fungus is a mycorrhizal fungus remains inconclusive [4,5].

**Figure 1:**
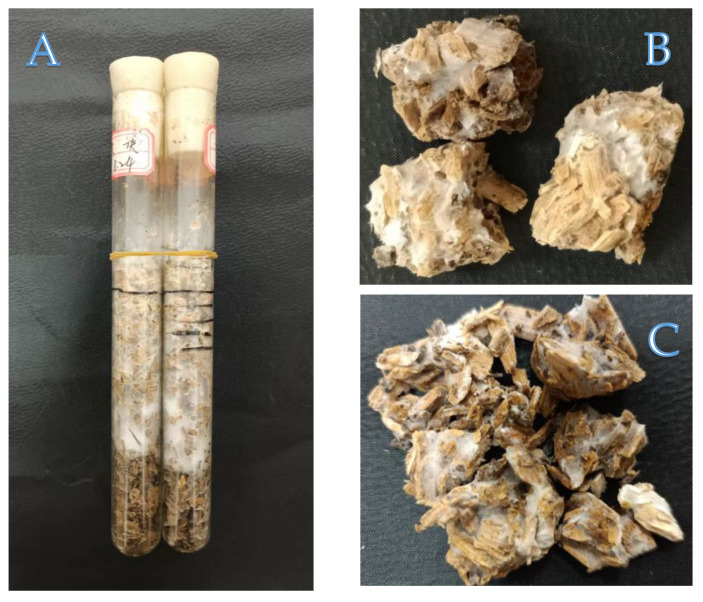
Experimental images of *Floccularia luteovirens* colonizing and growing on sawdust-based substrates (our research team). (**A**): Mycelia of *F. luteovirens* grew well in the sawdust-based substrate, with dense mycelia uniformly distributed throughout the substrate. (**B**): Substrate colonization performance of *F. luteovirens* mycelia: part of the sawdust was wrapped and covered by mycelia. (**C**): Substrate colonization performance of *F. luteovirens* mycelia: a large amount of sawdust was fully colonized and decomposed by mycelia.

Due to the constraints of traditional prior understanding, research on the domestication and cultivation of this fungus has been scarce. However, given its multiple aspects of significant value, achieving artificial cultivation is an inevitable trend, and our research team is currently conducting studies on the artificial domestication and cultivation of *F*. *luteovirens*. Based on preliminary observations from the exploratory experiments conducted by our research group, *F. luteovirens* can colonize and grow vigorously on sawdust-based substrates (Figure 1). This phenomenon suggests potential saprobic growth capacity under axenic conditions, prompting us to rethink: the classification of *F. luteovirens* as a mycorrhizal fungus is merely based on indirect evidence of “growth correlation with *Kobresia”*. Early studies inferred this symbiotic relationship through observations of hyphal attachment to *Kobresia* roots and the spatial association between fruiting bodies and plants, yet none provided morphological verification of typical mycorrhizal structures such as Hartig nets or mantles, nor did they offer molecular evidence of reciprocal interactions.

Meanwhile, our exploratory experiments demonstrated that this fungus can colonize and thrive on sawdust-based substrates. (The inoculum was derived from a sporocarp-isolated strain that was confirmed via ITS sequencing, with the strain number S4-1; the specific formulation is as follows: 73% mixed sawdust, 25% corn flour, 1% calcium superphosphate, and 1% gypsum; sterilization was carried out at 121 °C and 0.15 MPa for 1.5 h; each test tube was inoculated with four mycelial plugs, with four replicates per treatment, followed by incubation at a constant temperature of 25 °C in the dark; and the corresponding mycelial growth rate reached 0.958 mm/d after 30 days of cultivation). This differs from the traditional view that “mycorrhizal fungi depend on host symbiosis for survival.” It is noteworthy that many fungi capable of forming “fairy rings” inherently possess the strong capability to degrade soil organic matter (saprobic habits). Based on these findings, we propose the core hypothesis of “facultative nutrition”: *F. luteovirens* may possess the ability to independently degrade lignocellulose and acquire nutrients through a saprobic mode. This perspective challenges the traditional view that *F*. *luteovirens* must rely on mycorrhizal symbiosis, thereby opening up a completely new path for its artificial domestication.

The “facultative nutrition” strategy proposed in this study is more inclined to indicate that *F*. *luteovirens*, a species presumably classified as an ectomycorrhizal fungus, may possess the “facultative saprotrophic” capability to autonomously degrade dead organic matter. This is inherently distinct from the obligate mycorrhizal nutritional mode, which requires symbiosis with living plants for survival. However, the possibility of weak or conditional symbiosis in natural environments (i.e., “facultative symbiosis”) is not excluded, and the latter awaits direct verification through future research. The hypothesis and evidence chain presented in this study primarily support its potential for “facultative saprotrophy.”

## 2. Supporting Evidence for the Facultative Nutrition Hypothesis

The proposal of the “facultative nutrition” strategy for *F*. *luteovirens* is not based on a single experimental phenomenon, but rather, derived from a systematic collation of the evidence chain regarding its nutritional type—with the evidential flaws in its mycorrhizal fungal identity serving as the logical premise and multiple positive lines of evidence for its saprobic potential providing core support—and it is more consistent with the evolutionary rules of fungal nutritional types.

### 2.1. Review and Critique of Studies on Traditional Cognition

Over the years, the academic community has conducted several targeted studies on the core question of whether *F*. *luteovirens* is a mycorrhizal fungus. However, none of the relevant conclusions are supported by direct and robust mycorrhizological evidence. As early as 1997, Diao [6] first proposed the speculation that *F. luteovirens* forms a mycorrhizal relationship with *Kobresia*. Subsequently, the results of field habitat surveys by Li et al. [7] were basically consistent with Diao’s findings, further classifying it as an ectomycorrhizal fungus. In 2006, Lu et al. [8] conducted supplementary investigations on the vegetation types and companion plants of *F. luteovirens*, supporting the conclusion of a “mycorrhizal relationship.” In addition, Yang [9] and Ge et al. [10] directly cited previous conclusions in their related studies, stating that “ *F. luteovirens* is a mycorrhizal fungus symbiotic with forage grasses,” and thus argued that its artificial domestication and cultivation are extremely difficult.

However, all the aforementioned studies share a critical flaw: the lack of core evidence—none have provided morphological verification of typical mycorrhizal symbiotic structures such as Hartig nets, or offered direct molecular evidence. Conclusions are solely based on phenotypic observations and inferences from indirect correlations. To address this research gap, Wang et al. [11] conducted the first specialized verification study on the mycorrhizal relationship between *F. luteovirens* and plants. Ultimately, they failed to confirm the existence of a mycorrhizal relationship, only clarifying that there is a correlation between *Kobresia* and the growth and development of *F. luteovirens*.

In summary, none of the existing studies can directly and robustly prove that *F. luteovirens* is a mycorrhizal fungus. The conclusion of “mycorrhizal symbiosis” relies primarily on indirect inferences, with direct evidence remaining insufficient (Table 1). This finding provides a crucial premise for re-examining its nutrient acquisition strategy—since its mycorrhizal nature has not been confirmed, the possibility that it possesses saprobic or facultative nutritional capabilities increases significantly. The evidence related to wood-decaying potential discovered by our research team forms a precise logical correspondence with the evidential flaws in the theory of a mycorrhizal relationship, providing important reverse support for revealing the true nutritional type of *F. luteovirens*.

### 2.2. Positive Supporting Evidence for Saprobic Potential

Gan et al. [12] (2022) clarified the evolutionary relationship between *F. luteovirens* and *Agaricus bisporus* based on transcriptome sequencing and homology alignment. They found that the two fungi share the highest protein sequence homology, with the highest matching ratio (20.32%) occurring for *Agaricus bisporus* var. bisporus strain H97, followed by *Agaricus bisporus* var. burnettii strain JB137-S8 (17.25%). This result indicates that at the molecular evolutionary level, *F. luteovirens* has the closest genetic distance to *A. bisporus* (especially strain H97) and belongs to a closely related group. Furthermore, genome evolutionary analysis confirmed that *F. luteovirens* is “phylogenetically close to *A. bisporus*” in taxonomic status, and the two may share some basic metabolic pathways (e.g., purine metabolism, amino acid metabolism).

**Figure 2:**
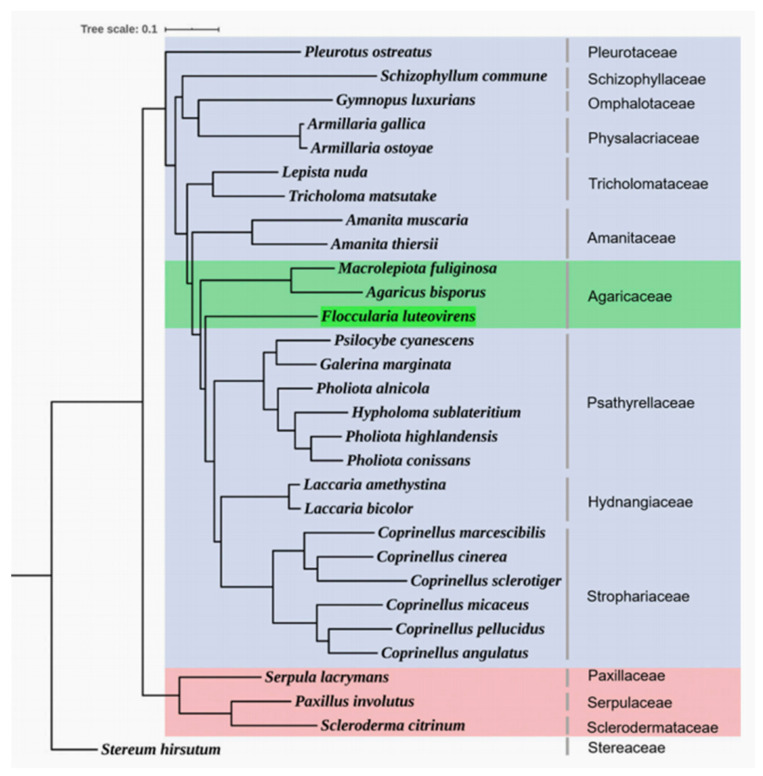
Phylogenetic tree indicating that *Floccularia luteovirens* and *Agaricus bisporus* are closely related groups (green part) (Gan et al., 2020); reprinted with permission from [13], Copyright 2020, Genetics Society of America. The fungus in Agaricales and other orders were colored in blue and pink, respectively. Among the Agaricales, the Agar-icaceae fungi was colored in green.

Gan et al. [13] (2020) focused on the genome of *F. luteovirens* and clarified its phylogenetic relationship with *A. bisporus* through multi-dimensional analysis as follows: (1) its gene structural characteristics (exon/intron length) are similar to those of *A. bisporus*; (2) the distribution pattern of repetitive sequences in the whole genome shares group-specific commonalities with *A. bisporus* (both take LTRs as the main transposon type); (3) its gene annotation coverage is close to that of *A. bisporus*; (4) a phylogenetic tree constructed based on single-copy genes from 30 basidiomycete species showed that *F. luteovirens* and *A. bisporus* belong to the same family (Agaricaceae) and are closely related to each other (Figure 2); (5) the proportion of orthologous genes shared with *A. bisporus* is the highest, further confirming their close phylogenetic relationship.

**Figure 3:**
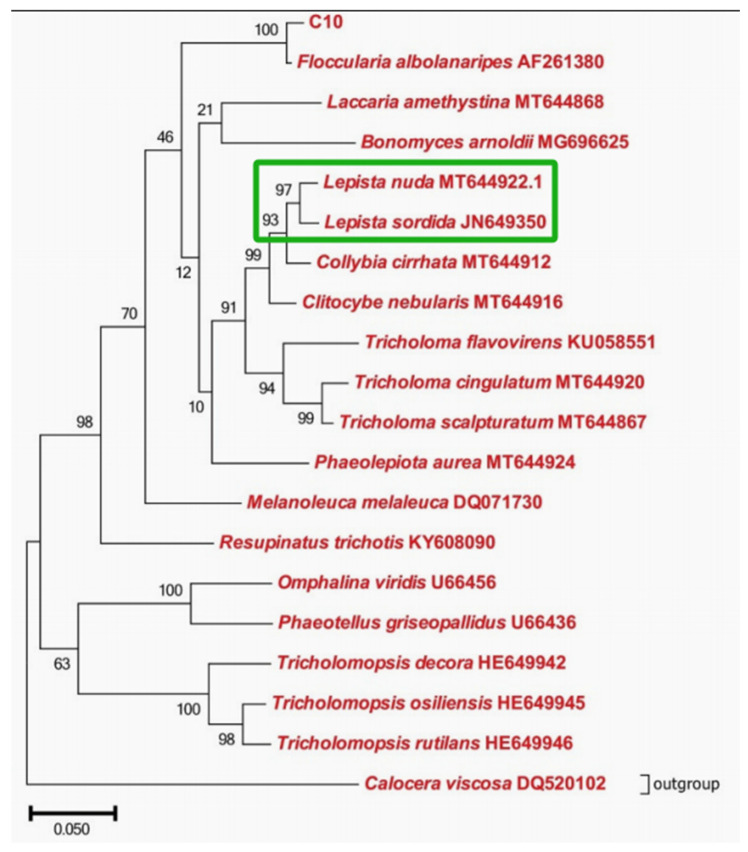
Phylogenetic tree showing that *Floccularia luteovirens* and *Lepista sordida* are closely related groups (green box) (Liu et al., 2021); adapted with permission from [14]. Copyright 2021, MDPI.

Liu et al. [14] clarified that *F*. *luteovirens* and *Lepista sordida* [15] are closely related groups (Figure 3) through phylogenetic analyses based on ITS sequences, LSU sequences, and whole-genome protein homology, with even higher ITS sequence homology. Most of the orthologous genes between the two are conserved basic metabolic genes (e.g., carbohydrate metabolism and amino acid transport genes), supporting their evolutionary affinity at the functional gene level. Through genome synteny analysis, the two exhibited the highest synteny, with longer and more continuous syntenic blocks—i.e., a large number of conserved fragments with “sequence similarity > 90% and length > 1 kb” exist in their genomes. In particular, the gene arrangement order related to basic metabolic pathways (e.g., glycolysis, ribosome biogenesis) is highly conserved, further verifying their close phylogenetic relationship.

Both Gan [13] and Liu [16] identified that *F*. *luteovirens* contains 365–400 carbohydrate-active-enzyme (CAZyme)-encoding genes, accounting for 8.58% of the total predicted protein-coding genes. These enzymes form the key enzyme system for its nutrient acquisition and are classified into six families: (1) glycoside hydrolases (GHs), the most abundant, capable of degrading polysaccharides such as cellulose and hemicellulose; (2) glycosyltransferases (GTs), involved in cell wall polysaccharide synthesis; (3) polysaccharide lyases (PLs), which degrade acidic polysaccharides such as pectin; (4) carbohydrate esterases (CEs), which hydrolyze carbohydrate ester bonds to enhance substrate degradation efficiency; (5) auxiliary activities (AAs), which assist in lignin degradation, and mostly include oxidoreductases; (6) carbohydrate-binding modules (CBMs), which anchor substrates without catalytic activity. Except for CBMs, the number of all CAZymes in *F. luteovirens* is higher than that in *Agaricus bisporus*. In addition, both Liu [16] and Ni [17] detected laccase (AA1 family, a key enzyme for lignocellulose degradation) in *F. luteovirens*. However, there are currently no research reports regarding the presence and activity levels of other key lignin-degrading enzymes, such as manganese peroxidase (MnP) and lignin peroxidase (LiP). The results for laccase detection indicate that *F*. *luteovirens* possesses preliminary lignin-modifying potential. Nevertheless, its complete lignin-degrading capability—particularly whether it harbors a diverse array of lignin-degrading enzyme systems—remains to be systematically verified through approaches such as targeted proteomics or specific enzyme activity assays.

Phylogenetically, the closely related taxa of *F*. *luteovirens* clearly include typical saprotrophic fungi, such as *Agaricus bisporus* [18] and *Lepista sordida* [19]. Although fungal trophic modes may exhibit a certain degree of phylogenetic conservatism [20], and species within the same family or genus sometimes share similar nutritional strategies [21], it must be pointed out that trophic modes are not strictly conserved. Cases of trophic shifts among related taxa have also been reported in basidiomycetes [22,23]. Therefore, the saprotrophic habits of closely related taxa provide valuable phylogenetic context clues and a plausible evolutionary scenario for the potential saprotrophic capability of *F. luteovirens*, but these cannot serve as definitive evidence that it necessarily inherits the metabolic basis for saprotrophy. This suggests that *F. luteovirens* may inherit saprobic-related metabolic foundations. Furthermore, enzymatic activity provides enzymatic evidence for its saprobic potential. From a nutrient utilization perspective, Guo [24] observed significant differences in the growth of *F. luteovirens* mycelia between media using living plants as carbon sources and those using dead carbon sources (e.g., sucrose): with PDA medium as the basal medium, adding 10% juice of *Kobresia* humilis to PDA resulted in significantly better growth of *F. luteovirens* mycelia on the grass juice medium than on PDA (*p* < 0.05). This result indicates that *F. luteovirens* can effectively utilize nutrients in grass juice, but it does not directly prove a symbiotic relationship with living *Kobresia* plants—consistent with the “facultative nutrition” hypothesis that “multiple nutrient sources can be utilized.”

In summary, these studies reflect significant gaps in the evidence chain defining *F. luteovirens* as a mycorrhizal fungus. Its nutritional type may possess greater plasticity or diversity than traditionally recognized. This provides the necessary logical premise and exploration space for the “facultative nutrition” hypothesis proposed in this paper.

## 3. Lines of Evidence Supporting Saprobic Potential

### 3.1. Enzyme System Comparison

The composition and content of carbohydrate-active enzymes (CAZymes) in fungi are closely related to their lifestyles [25,26]. Previous studies have shown that the types and quantities of fungal CAZymes can reflect fungi’s adaptation to and preference for different types of plant biomass and lifestyles [27]. *Agaricus bisporus*, a fungus that decomposes plant residues, typically possesses abundant CAZyme families such as cellulases (e.g., GH6, GH7), hemicellulases (e.g., GH10, GH11), and pectinases (e.g., PL1, PL3) to efficiently degrade lignocellulose for carbon source acquisition [28,29]. In contrast, obligate ectomycorrhizal fungi such as *Boletus edulis* have lost most plant cell wall-degrading enzymes due to their dependence on host photosynthates during symbiosis with host plants, and their CAZyme profiles exhibit obvious “degenerative” characteristics [30,31]. Therefore, comparing the CAZyme family composition of fungi with different nutritional types can effectively infer their ecological strategies and degradation potential at the enzymatic level.

To verify whether *F*. *luteovirens* possesses lignocellulose degradation potential characteristic of facultative nutritional fungi, we selected the typical saprobic fungus *Agaricus bisporus*, its closely related species *Lepista sordida* [32], and the typical ectomycorrhizal fungus *Boletus edulis* [33,34] as controls, and systematically compared the compositional differences in their key CAZyme families (Table 2).

This comparison suggests that *F. luteovirens* appears to retain multiple key plant cell-wall-degrading enzyme families (e.g., GH6, GH7, GH10, PL1), with some families (e.g., GH6, GH7) reported to exhibit an expansion trend. The CAZyme profile of *F. luteovirens* is broadly consistent with that of *Agaricus bisporus*, while differing notably from that of *Boletus edulis*.

It should be noted that the analysis of the CAZyme profile of *F. luteovirens* in this study is primarily based on genome annotation and qualitative “presence/absence” comparisons with the existing literature (as shown in Table 2). More refined bioinformatic validations, such as expression quantification, secretome prediction, or phylogenetic principal component analysis (PCA), have not yet been conducted. Although the current data clearly indicate the presence of key degradative enzymes such as GH6, GH7, and AA1, and the profile characteristics are similar to those of saprophytic fungi, this comparison remains preliminary. Future studies should integrate transcriptomics, proteomics, and secreted protein prediction to further validate the activity and function of these CAZymes in actual degradation processes.

### 3.2. Nutritional Utilization Characteristics of Closely Related Species

A study by Takano et al. [35] demonstrated that *Lepista sordida* (the sordid blewit) has been identified as a basidiomycete with white-rot characteristics. This conclusion is mainly based on the complete lignocellulose-degrading enzyme system encoded in its genome, particularly the abundant enzymes belonging to the auxiliary activity (AA) family. Specifically, a variety of typical enzyme genes characteristic of white-rot fungi have been identified in the genome of *Lepista sordida*, including manganese peroxidase (MnP), laccase (Lac) and cellobiose dehydrogenase (CDH); these enzymes together form a highly efficient molecular machinery for lignin degradation. In addition, functional annotation of the genome revealed a complete carbohydrate-active enzyme (CAZy) system, which covers multiple families such as glycoside hydrolases (GHs), polysaccharide lyases (PLs), carbohydrate esterases (CEs) and carbohydrate-binding modules (CBMs). This multi-enzyme synergistic mode of action is a typical molecular feature of white-rot fungi in lignocellulose degradation. Comparative genomic analysis further indicated that *Lepista sordida* shares a high degree of conservation in the key lignin-degrading genes with well-characterized white-rot fungi (e.g., *Lentinula edodes*). Alim et al. [36] indicated that this species grows in soil or humus, predominantly on the ground or leaf litter rather than on wood, thus exhibiting saprobic properties. As a closely related species, existing artificial cultivation studies on *Lepista sordida* have confirmed its adaptability to various lignocellulosic substrates [21,37,38,39,40,41,42,43,44,45,46,47]. Multiple cultivation studies (detailed information is summarized in Supplementary Table S2) have demonstrated that *Lepista sordida* can effectively utilize a variety of agricultural and forestry wastes—including rice straw, corncobs, soybean straw, tea tree sawdust, and even treated pine and fir sawdust—to complete its entire life cycle from mycelial colonization to fruiting body formation. As a closely related species of *F. luteovirens*, the successful cultivation practice of *Lepista sordida* provides important physiological and ecological circumstantial evidence that fungi of the same genus or closely related groups may share similar nutritional adaptability. However, it must be recognized that there is a possibility of niche differentiation between different species. Therefore, this evidence mainly serves as a suggestion and a reference, and the saprobic capacity of *F. luteovirens* itself still needs to be confirmed through direct cultivation experiments.

As can be seen from the research results in the table, *Lepista sordida* can not only utilize common agricultural wastes such as rice straw and corn cob, but also adapt to complex lignocellulosic substrates including tea tree sawdust, cherry branches and even treated pine and fir sawdust. These cultivation experiments generally achieved the complete life cycle from rapid mycelial colonization to successful fruiting body formation, fully demonstrating that *Lepista sordida* possesses a robust and efficient saprobic catabolic system, which enables it to independently obtain nutrients from non-living plant materials for growth and development. Considering the close genetic relationship between *F. luteovirens* and *Lepista sordida*, this experimentally verified saprobic capability that can be realized across multiple substrates provides strong indirect support for inferring that *F. luteovirens* may possess similar saprobic potential. Therefore, in addition to genomic CAZyme analysis, the evidence chain from the successful domestication and cultivation of closely related species provides valuable clues and a low-risk starting point for further research on the facultative nutritional potential of *F. luteovirens*.

Integrating genomic CAZyme profiles with cultivation practices of closely related species, *F. luteovirens* exhibits degradation potential similar to typical facultative nutrient fungi. The key enzyme families it retains—such as GH6, GH7, and PL1—show high similarity to those of “transitional fungi” (e.g., certain species in the Agaricaceae family) capable of switching between saprophytic and endophytic modes. These enzymatic features not only support its ability to degrade lignocellulose but also suggest its potential to flexibly adjust nutrient strategies across different ecological niches (e.g., humus-rich environments or plant rhizospheres). For instance, it may activate saprophytic pathways under conditions rich in non-living organic matter, while potentially utilizing weak symbiotic signals to aid with colonization in living rhizospheric environments. This characteristic starkly contrasts with obligate mycorrhizal fungi (such as Boletus edulis), which strictly depend on host plants, further supporting the rationality of the “facultative nutrient” hypothesis.

## 4. Reference Insights for Domestication and Cultivation Substrates of *F. luteovirens*

Based on the mature practice of cultivating *Lepista sordida* on sawdust-based substrates (Supplementary Table S2), combined with the phenomenon of good colonization of *F. luteovirens* on sawdust substrates observed by our research group (Figure 1), this study holds that the observations made during the cultivation of *Lepista sordida* can provide a reference for the direction of the domestication of *F. luteovirens*. However, the specific formulas and processes still need to be optimized and verified according to the biological characteristics of *F. luteovirens.*

### 4.1. Sawdust-Based Substrates Are the Priority for Domestication and Cultivation

Cultivation studies on *Lepista sordida* have systematically verified the universal adaptability of sawdust as a core substrate: the full growth cycle of *Lepista sordida*, including mycelial colonization, primordium formation and fruiting body development, can be completed on broadleaf sawdust, tea tree sawdust, and even treated coniferous sawdust, with a biological efficiency ranging from 51.38% to 68.3% (Supplementary Table S2). This indicates that the lignocellulosic components contained in sawdust can effectively meet the nutritional requirements of this closely related saprobic fungus. Given the high similarity in CAZyme profiles between *F. luteovirens* and *Lepista sordida* (Table 2), and the vigorous mycelial growth of *F. luteovirens* on sawdust substrates observed in our preliminary experiments (Figure 1), sawdust-based substrates can be directly used as the preferred raw materials for its domestication and cultivation, and provides a rational starting point.

### 4.2. Substrate Optimization Should Balance Growth Efficiency and Fruiting Body Quality

According to the cultivation effects of *Lepista sordida*, the following priority ranking for selecting sawdust substrates for *F. luteovirens* is proposed:

First choice for basic domestication: broadleaf sawdust as the core conventional substrate. All seven broadleaf sawdust-based composite formulations were suitable for the growth of *Lepista sordida* spawn, with the optimal formulation achieving a mycelial growth rate of 0.8 cm/d and a full-bag time of only 35 days (Supplementary Table S2). It can serve as the basic sawdust substrate for the domestication of *F. luteovirens*, ensuring robust mycelial growth and stable fruiting.

First choice for domestication optimization: tea tree sawdust or fermented tea branch substrate. When *Lepista sordida* was cultivated on such substrates, the crude protein content of fruiting bodies increased to 21.3% and polysaccharide content reached 3.2%, with the mycelial colonization period shortened by 3–5 days (Supplementary Table S2). The application of such substrates in the domestication of *F. luteovirens* may simultaneously optimize yield and nutritional quality.

Option for sustainable utilization: fermented coniferous sawdust (pine and fir sawdust). Studies on *Lepista sordida* have confirmed that pretreatment can eliminate inhibitory substances, resulting in a yield with no significant difference compared with broadleaf sawdust substrate (Supplementary Table S2). This substrate can be used to expand the raw material sources for *F. luteovirens* domestication, but corresponding pretreatment processes are required.

### 4.3. Value of Insights

By integrating empirical cultivation data of closely related species, this section provides a low-trial-and-error-cost pathway for the domestication of *F. luteovirens*. It not only further corroborates the facultative nutritional metabolic potential of *F. luteovirens* (which matches its CAZyme profile), but also lays a technical foundation for realizing large-scale and high-quality artificial cultivation. Future research can build on this basis to conduct refined optimization of specific regulatory factors for *F. luteovirens* (e.g., pH, humidity).

New Insights into the Ecological Role of the “Fairy Rings” Formed by *F. luteovirens*: Traditionally, the formation of fairy rings by *F. luteovirens* has been attributed to nutrient exchange under mycorrhizal symbiosis. However, the evidence provided in this study indicates that *F. luteovirens* may possess significant lignocellulose-degrading potential. This offers an alternative explanation for the fairy ring phenomenon: the formation of fairy rings might be associated with the intense decomposing activity of its mycelial front on dead organic matter (such as grass roots) in the soil, thereby leading to differences in grass growth inside and outside the rings. This ecological role centered on saprobism is more similar to that of many known saprobic fairy-ring-forming fungi (e.g., certain species of *Lepiota*). Future research should focus on distinguishing the relative contributions of these two mechanisms (symbiosis vs. saprobism) to fairy ring formation through in situ experiments.

## 5. Prospects

### 5.1. In-Depth Analysis of the Molecular Mechanisms Underlying the Nutritional Type of F. luteovirens

Recent genomic and CAZyme profile analyses have demonstrated that *F. luteovirens* possesses a nearly complete lignocellulose-degrading toolkit (Table 2), indicating its robust saprobic capabilities. However, several core issues remain to be addressed: whether these degradative enzyme genes are truly activated in vivo, how their expression regulatory networks respond to different carbon sources (e.g., living plant root exudates vs. dead lignocellulose), and whether their functions can be verified at the protein and enzymatic activity levels.

Future research should focus on the following three aspects:

(1) Time-series multi-omics analysis: Establish culture systems with different nutrient sources (e.g., glucose, *Kobresia* root extracts, straw lignocellulose) under strictly controlled conditions, and perform dynamic monitoring of the transcriptome, proteome, and metabolome of *F. luteovirens*. This will directly reveal its carbon source utilization preferences and the activation timing of key degradation pathways.
(2) Systematic prediction of CAZyme expression and secreted proteins: It is recommended to use secretome prediction tools (e.g., SignalP, TargetP) to identify secretion signal peptides in the CAZyme-encoding genes of *F. luteovirens*. Combined with comparative genomics, CAZyme heatmaps should be constructed and PCA conducted comparing *F. luteovirens* with typical saprophytic and mycorrhizal fungi to more intuitively reveal the evolutionary positioning of its enzyme profile and degradation strategies. Such analyses will help clarify the actual secretory capacity and ecological functional division of its degradation enzyme system.
(3) Functional validation of key CAZyme genes: Conduct loss-of-function/gain-of-function studies on critical degradative enzyme genes such as GH6, GH7, and AA1 using techniques including gene knockout, RNA interference (RNAi), and overexpression, to directly verify their necessity in the lignocellulose degradation process.
(4) Exploration of symbiosis-related signaling pathways: Despite the lack of direct evidence, the possibility of weak interactions between *F. luteovirens* and *Kobresia* in natural habitats cannot be completely ruled out. A co-culture system should be employed to detect the induced expression of mycorrhizal symbiosis marker genes (e.g., mycorrhiza-induced small secreted proteins, MISSPs), thereby clarifying whether the fungus retains basic symbiotic “communication” capabilities. This study is crucial for accurately defining the nutritional type of *F. luteovirens*—whether it leans toward “facultative saprotrophy” (an ectomycorrhizal [ECM] fungus acquiring saprobic capabilities) or “facultative symbiosis” (a fungus not relying on a specific host)—which will deepen the understanding of its ecological adaptation strategies.

### 5.2. Field Validation of the Ecological Significance of Facultative Nutrition and Research on F. luteovirens’ Regulatory Mechanisms

The proposal of the “facultative nutrition” hypothesis implies that *F. luteovirens* may switch its nutritional strategies according to environmental conditions. Future work should carry out the following to extend from laboratory studies to field investigations to validate its ecological significance and elucidate the underlying regulatory mechanisms.

In situ field monitoring: Combine stable isotope labeling (e.g., ^13^CO_2_ pulse labeling of *Kobresia*) with high-throughput sequencing technology to track the flow direction and allocation efficiency of photosynthetic carbon in the *Kobresia*–*F. luteovirens* system, directly verifying whether the fungus can acquire carbon sources from host plants under natural conditions.

Analysis of environmental factor regulatory networks: Focus on investigating how key environmental factors such as temperature, moisture, and nitrogen–phosphorus nutrients regulate the “switch” of *F. luteovirens’* nutritional strategies. For example, under nutrient stress, does it tend to activate the saprobic pathway? This requires constructing a response model between its nutritional strategies and environmental factors through controlled microcosm experiments.

Population genetic analysis: Perform whole-genome re-sequencing of *F. luteovirens* populations from different geographical locations, analyze the genetic diversity and selection signatures of CAZyme gene families and symbiosis-related genes, and understand the shaping effect of environmental pressures on its nutritional strategies from an evolutionary perspective.

### 5.3. Optimization of the Artificial Domestication and Cultivation Technology System Based on Facultative Nutrition Strategies

This study has laid a solid theoretical foundation for the domestication of *F. luteovirens* and confirmed the feasibility of using sawdust-based substrates as core cultivation materials. Subsequent applied research should focus on the refinement and standardization of the technical system by carrying out the following.

Precise optimization of dedicated cultivation substrate formulations: On the basis of confirming sawdust as the main material, systematically evaluate the effects of different sawdust types (broadleaf vs. coniferous), particle sizes, carbon–nitrogen ratios, and additive amounts of auxiliary materials (e.g., wheat bran, corn flour) on mycelial growth, primordium formation, fruiting body yield, and quality (flavor substances, active ingredients), and establish the optimal formulation.

Intelligent regulation of cultivation environment factors: Clarify the precise requirements for temperature, humidity, light, and CO_2_ concentration during each stage of mycelial growth, primordium differentiation, and fruiting body development, and develop corresponding intelligent environmental control systems to achieve high-yield, high-quality, and year-round production.

In addition, future research should focus on integrating bioinformatics with experimental validation. Through methods such as secretome prediction and co-expression network analysis, the regulatory patterns of CAZyme genes in *F. luteovirens* under different nutritional conditions should be systematically elucidated, thereby providing a more comprehensive evidence chain for the molecular mechanisms of its facultative nutrient strategy.

### 5.4. Resource Conservation and Sustainable Utilization

As a rare endemic resource of the Qinghai–Tibet Plateau, the artificial domestication of *F. luteovirens* must be accompanied by the placing of high priority on the protection of wild resources. This can be achieved by carrying out the following:

Establishment and evaluation of germplasm resource banks: Systematically collect and preserve wild germplasm resources from different geographical populations, and conduct comprehensive evaluations of agronomic traits (e.g., temperature type, fruiting period, yield), quality traits, and stress resistance, to provide material support for the breeding of superior strains.

Promotion of in situ conservation through artificial cultivation: Successful artificial domestication will greatly alleviate the harvesting pressure on wild resources. A “bionic cultivation” model (e.g., semi-artificial cultivation in the original habitat) can be adopted to achieve a win–win situation of industrial development and ecological protection.

In summary, the renewed understanding of the nutritional type of *F. luteovirens*—“facultative nutrition”—has opened a new door for basic biological research on this species and its industrial development. Future research should be systematically advanced along the path of “deciphering molecular mechanisms → clarifying ecological rules → optimizing cultivation technologies → realizing sustainable resource utilization”. Ultimately, this alpine delicacy will be able to be distributed beyond the alpine meadows and achieve large-scale and standardized production, contributing to the development of plateau-characteristic agriculture, an increase in herdsmen’s income, and the protection of biodiversity.

## Figures and Tables

**Table 1:** Collation and critical analysis of evidence from studies related to the mycorrhizal relationship of *Floccularia luteovirens* with plants.

Researchers	Research Content	Findings /Conclusions	Indirect Supporting Evidence	Missing Direct Evidence/Critical Points	Proposed Confirmatory Molecular Evidence	References
Diao Zhimin	Survey of vegetation in the occurrence area of *F*. *luteovirens* and analysis of its relationship with *Kobresia.*	*F. luteovirens* forms a mycorrhizal relationship with *Kobresia*.	1. *Kobresia* in the growth area grew well (dark green and lush). 2. *Kobresia* around the fairy rings turned yellow early during the fruiting body growth season. 3. White hyphae were attached to the underground stems of *Kobresia*.	No direct mycorrhizological evidence was provided; the existence of typical symbiotic structures such as Hartig nets was not verified, and the conclusion was only inferred based on indirect phenotypic observations and hyphal attachment phenomena.	Dual RNA sequencing of the plant–fungal symbiotic system should be adopted to specifically verify whether the symbiosis marker genes of *F*. *luteovirens* (e.g., MiSSPs) are induced and expressed at the rhizosphere interface. Combined with stable isotope labeling, the efficiency of plant photosynthetic carbon transfer to the fungus should be quantified to functionally rule out the possibility of obligate symbiosis.	[6]
Li et al.	Field habitat survey of *F*. *luteovirens* and analysis of its association with plant roots.	*F. luteovirens* appears to form ectomycorrhizae with *Kobresia/Carex* and is classified as an ectomycorrhizal fungus.	This study’s results are basically consistent with Diao’s research findings, inferred based on the spatial association between the occurrence site of fruiting bodies and plant roots.	Also lacks morphological or molecular evidence of direct mycorrhizal structures (e.g., Hartig net), and the designation of “ectomycorrhizal fungus” lacks verification of core symbiotic structures.		[7]
Lu et al.	Survey of vegetation types and companion plants of *F*. *luteovirens.*	*F. luteovirens* forms a mycorrhizal relationship with its companion plant (*Kobresia*).	1. Mainly grows in alpine meadow and alpine steppe vegetation. 2. Hyphae are attached to the roots of *Kobresia.*	Morphological identification of mycorrhizae (e.g., Hartig net, mantle) was not conducted; the conclusion is only based on indirect inference from hyphal attachment phenomena, without support from direct symbiotic evidence.		[8]
Yang et al.	Analysis of the ecological habits and feasibility of artificial domestication of *F*. *luteovirens.*	*F. luteovirens* is a mycorrhizal fungus symbiotic with forage grasses, and its artificial domestication is extremely difficult.	Based on the citation and extension of previous research conclusions; no independent verification experiments were conducted.	Lacks support from independent research data; the conclusion relies on indirect inferences, and fails to address the core issue that “the mycorrhizal relationship lacks direct evidence”.		[9]
Ge et al.	Analysis of the ecological habits and feasibility of artificial domestication of *F*. *luteovirens.*	*F. luteovirens* is a mycorrhizal fungus symbiotic with forage grasses, and its artificial domestication is extremely difficult.	Based on the citation and extension of previous research conclusions; no independent verification experiments were conducted.	Lacks support from independent research data; the conclusion relies on indirect inferences, and fails to address the core issue that “the mycorrhizal relationship lacks direct evidence”.		[10]
Wang et al.	Targeted verification of the mycorrhizal relationship between *F*. *luteovirens* and plants (*Kobresia*) (first specialized study).	Failed to find sufficient evidence to confirm the mycorrhizal relationship, only indicating a correlation between *Kobresia* and the growth and development of *F. luteovirens*.	1. AM fungal colonization was observed in the roots of *Kobresia*. 2. Gel electrophoresis failed to detect amplified bands related to *F. luteovirens*.	1. It was not clarified whether the AM fungi colonizing the roots were *F. luteovirens*. 2. There were potential confounding factors such as insufficient sample size and mismatch between sampling time and the mycorrhizal formation period. 3. The existence of typical mycorrhizal structures was still not verified.	It is recommended to use *F*. *luteovirens*-specific molecular markers for in situ hybridization to clarify the identity of rhizospheric hyphae. Additionally, controlled carbon source competition experiments should be conducted to compare its utilization preference between plant photosynthetic carbon and soil organic carbon, thereby providing direct physiological and ecological evidence for excluding its nutritionally dependent symbiotic relationship.	[11]

**Table 2:** Schematic literature-based comparison.

CAZyme Family	Functional Description	*Floccularia luteovirens*	*Agaricus bisporus*	*Lepista sordida*	*Boletus edulis*
Cellulose Degradation-Related					
GH6	Cellobiohydrolase (CBH II), attacks the non-reducing end of cellulose	Present (expanded) [13,16]	Present (present in white-rot ancestor) [29]	Present (genomically encoded) [32]	Absent/significantly reduced (typical ECM characteristic) [33,34]
GH7	Cellobiohydrolase (CBH I), attacks the reducing end of cellulose	Present (expanded) [13,16]	Present [29]	Present [32]	Absent/significantly reduced [33,34]
AA9 (LPMO)	Lytic Polysaccharide Monooxygenase (LPMO), oxidatively cleaves crystalline cellulose	Present (multiple copies) [13,16]	Present (abundant) [29]	Present [32]	Present but in low abundance [33]
Hemicellulose Degradation-Related					
GH10	Endoxylanase (degrades the xylan backbone)	Present [13,16]	Present (abundant) [29]	Present [32]	Reduced [34]
GH11	Endoxylanase	Present	Present [29]	Present	Reduced/absent
GH43	β-xylosidase/α-arabinofuranosidase, etc.	Present [13,16]	Present [29]	Present [32]	Reduced [34]
Pectin Degradation-Related					
PL1	Pectin Lyase;	Present [13,16]	Present [29]	Present (related studies)	Usually absent/extremely rare (typical ECM characteristic) [33]
PL3	Pectin Lyase	Present [13,16]	Present [29]	Present	Usually absent/extremely rare [33]
GH28	Polygalacturonase/Pectin Hydrolase	Present [13,16]	Present [29]	Present [32]	Significantly reduced/absent [33]
Lignin Modification-Related					
AA2	Lignin Peroxidase (LiP)/Manganese Peroxidase (MnP)	Not clear yet	Present (abundant, with lignin-degrading capacity) [29]	Present (e.g., laccase) [32]	Significantly reduced/absent (depends on non-enzymatic mechanisms) [33]
AA1	Laccase	Present [13,16]	Present [29]	Present (activity studied) [32]	Present (yet its function tends to be involved in symbiotic interface formation rather than degradation) [33]
Overall CAZyme Abundance		High (the genome contains 7068 protein-coding genes with diverse CAZymes) [13,16]	High (adapted to saprobic lifestyle for degrading complex plant materials) [29]	High (endowed with lignocellulose-degrading capability) [32]	Low (genome reduction with massive loss of PCWDEs) [33,34]

## Data Availability

The data presented in this study are available in the article and its Supplementary Materials. This research utilized data from previously published studies as cited in the reference list. Project-specific preliminary data and related images are included in the main text.

## Supplementary Materials

The following supporting information can be downloaded at: https://www.mdpi.com/article/10.3390/life16020287/s1, Supplementary Table S1: Evidence Framework for Mycorrhizal Relationship Diagnosis of Floccularia luteovirens; Supplementary Table S2: Summary of Research Findings Related to the Cultivation of Lepista sordida.
