# Supplementary material for: Revisiting the Nutritional Mode of Floccularia luteovirens: A Case for Facultative Saprobic Capacity
Source: Life (Basel). 2026 Feb 7;16(2):287. doi: 10.3390/life16020287 (PMC12941505; doi:10.3390/life16020287)
Supplement: Supplementary file 1 [file life-16-00287-s001.zip › life-4113459-supplementary.pdf]

Supplementary Table S1

Evidence Framework for Mycorrhizal Relationship Diagnosis of *Floccularia luteovirens*

Study type	Sampling design	Fungal detection method	Mycorrhizal structure evidence	Limitations
Field ecological survey	Observing the vegetation distribution in *F. luteovirens* habitats and their spatial association with Kobresia	Morphological Observation (Root Mycelium)	Direct morphological evidence of typical symbiotic structures (Hartig net and mantle) is lacking	The conclusions are inferred based on indirect phenotypic observations (e.g., differences in grass vigor around fairy rings, mycelial attachment on roots) and lack core verification in mycorrhizal studies.
	Investigation of Spatial Association Between Fruiting Body Occurrence Sites and Plant Root Systems	Morphological Observation (Inference Based on Spatial Association)	No morphological or molecular evidence supports the presence of direct mycorrhizal structures such as the Hartig net	The designation of the fungus as an "ectomycorrhizal fungus" lacks verification of core symbiotic structures, resulting in a weak evidence chain
Survey of Vegetation and Associated Plants	Investigation of Vegetation Types and Associated Plants (Kobresia) for *F. luteovirens*	Morphological Observation (Root Mycelial Attachment)	No morphological identification of mycorrhizal structures (Hartig net and mantle) was conducted	Inference was solely based on mycelial attachment, lacking direct evidence for symbiosis
Analysis of Ecological Habits and Domestication Feasibility	This study is based on theoretical analysis through literature citation, without conducting independent field sampling or experiments	Not Applicable (Literature Review)	No new direct or indirect evidence has been provided	Lacking support from independently generated research data, the conclusions rely on indirect inferences drawn by previous studies and fail to address the core issue of insufficient evidence
Targeted Validation Study on Mycorrhizal Relationships (First Specialized Research)	Collect root samples of Kobresia to attempt verification of mycorrhizal associations	Morphological Observation (Detection of AM Fungal Colonization), Gel Electrophoresis (Target Bands Not Detected)	No typical ectomycorrhizal structures (such as the Hartig net) were confirmed. The identity of the root-colonizing AM fungi as *F. luteovirens* is unconfirmed	1.The methodology may be mismatched (Is an arbuscular mycorrhizal (AM) fungal indicator applied to ectomycorrhizal fungi?).<br>2.There exist potential confounding factors such as insufficient sample size and mismatches between sampling time and the mycorrhizal formation period.<br>3.Direct evidence supporting the mycorrhizal relationship has not yet been obtained.

Supplementary Table S2

Summary of Research Findings Related to the Cultivation of *Lepista sordida*

Researchers (Year)	Research Content	Results	Substrate Type	Key Data/Conclusions	References
Thongbai et al. (2017)	First artificial cultivation experiment of *Lepista sordida* in Thailand, exploring optimal cultivation conditions	Successfully obtained mature fruiting bodies and established a basic cultivation technical protocol	Rice straw	Primordia formed and developed into mature fruiting bodies 31 days after inoculation at 25–30 °C; Three flushes of fruiting were achieved, with the second flush occurring on days 38–44 and the final flush on days 45–52	[37]
Xu et al. (2021)	Optimization of artificial cultivation techniques for *Lepista sordida*, focusing on shortening the fruiting cycle and increasing fruiting body yield	Shortened the fruiting cycle and achieved stable fruiting	Corn cob	Mycelial colonization and casing soil adaptation were completed 20 days after inoculation, with the first flush of mature fruiting bodies harvestable on day 30; The second flush occurred on days 37–43 and the final flush on days 48–50	[38]
Sheng et al. (2024)	Comparing the effects of three agricultural wastes (rice straw, corn cob, soybean straw) on the growth, development and yield of *Lepista sordida*	Cultivation effects of corn cob and soybean straw substrates were significantly superior to those of rice straw substrate	Rice straw, corn cob, soybean straw	Comprehensive evaluation of fruiting body morphology, individual fruiting body weight and total yield showed that the biological efficiency of corn cob and soybean straw substrates increased by 15%–20% compared with rice straw substrate	[39]
Sheng et al. (2023)	Investigating the effect of spent *Auricularia* cornea substrate (SSA) as an alternative substrate on the cultivation of *Lepista sordida*	Addition of spent *Auricularia* cornea substrate significantly improved the total yield and biological efficiency of *Lepista sordida*	Corn straw (control group), spent *Auricularia* cornea substrate (alternative substrate group)	A total of three flushes of fruiting bodies were harvested; The total biological efficiency of the alternative substrate group reached 68.3%, which was 22.5% higher than that of the control group, realizing the resource utilization of agricultural wastes	[40]
Li H. et al. (2023)	Technical exploration of cultivating *Lepista sordida* on tea tree sawdust and nutritional component analysis of fruiting bodies	Tea tree sawdust can be used as a high-quality cultivation substrate for *Lepista sordida*, with nutrient-enriched fruiting bodies	Tea tree sawdust	The crude protein content of fruiting bodies reached 21.3% and polysaccharide content 3.2%, with abundant types of flavor substances, meeting the standards of high-quality edible fungi	[41]
Zhang X.W. et al. (2022)	Effects of fermented tea branch substrate on mycelial growth, colonization rate and fruiting body differentiation of *Lepista sordida*	Fermented tea branch substrate is suitable for the growth requirements of *Lepista sordida* and supports efficient fruiting	Fermented tea branch substrate	Mycelial germination rate reached 98%, the colonization cycle was shortened by 3–5 days compared with conventional substrates, and the uniformity of fruiting body differentiation was improved by 30%	[42]
He X.M. et al. (2022)	Study on understory cultivation technology of *Lepista sordida* using cherry branches as the main substrate	Successfully realized the resource utilization of cherry branches and filled the gap in understory cultivation technology of cherry orchards	Cherry branches (main substrate)	Under understory cultivation conditions, the survival rate of fruiting bodies reached 92%, and the total yield was 18% higher than that of open-field cultivation, achieving a win-win situation of ecological and economic benefits	[43]

Li B.Q. et al. (2014)	Screening of stock culture media for *Lepista sordida*, focusing on the optimization of sawdust-based formulations	Sawdust can be used as the core component of stock culture media for *Lepista sordida*	Sawdust (supplemented with wheat bran and other auxiliary materials)	The optimized sawdust-based formulation shortened the mycelial full-bottle time to 28 days, with robust mycelial growth and contamination rate controlled below 3%	[44]
Hu X.Y. et al. (2006)	Screening of spawn culture formulations for *Lepista sordida*, comparing the adaptability of different sawdust-based formulations	All formulations with sawdust as the main material can meet the growth requirements of spawn cultures	7 composite formulations with sawdust as the main material	The mycelial growth rate of the optimal formulation reached 0.8 cm/d, and the full-bag time was 35 days, providing technical support for large-scale cultivation	[45]
Yue W.S. et al. (2023)	Domestication and cultivation of wild strains of *Lepista sordida*, comparing the morphological and nutritional components of domesticated and wild fruiting bodies;	Domestication and cultivation were successful, with the morphological characteristics of domesticated fruiting bodies generally consistent with wild-type ones and nutrients well preserved	Composite domestication substrate of sawdust+cottonseed hulls	The differences in pileus diameter and stipe length between domesticated and wild fruiting bodies were less than 10%, and the contents of crude polysaccharides and amino acids were basically equivalent	[46]
Lu C.Y. et al. (1994)	Verification of the applicability of pine and fir sawdust for *Lepista sordida* cultivation, exploring the utilization potential of coniferous sawdust	Fermented pine and fir sawdust can be used as a conventional cultivation substrate for *Lepista sordida*	Fermented pine and fir sawdust (main substrate)	Fermentation treatment reduced the inhibitory substances in pine and fir sawdust, mycelial growth was normal, and there was no significant difference in fruiting body yield compared with the broadleaf sawdust substrate	[47]
